# False Data Injection Detection for Phasor Measurement Units

**DOI:** 10.3390/s22093146

**Published:** 2022-04-20

**Authors:** Saleh Almasabi, Turki Alsuwian, Muhammad Awais, Muhammad Irfan, Mohammed Jalalah, Belqasem Aljafari, Farid A. Harraz

**Affiliations:** 1 Electrical Engineering Department, College of Engineering, Najran University, Najran 11001, Saudi Arabia; ssalmasabi@nu.edu.sa (S.A.); miditta@nu.edu.sa (M.I.); msjalalah@nu.edu.sa (M.J.); bhaljafari@nu.edu.sa (B.A.); 2 Department of Computer Science, Edge Hill University, Ormskirk L39 4QP, UK; awaism@edgehill.ac.uk; 3 Promising Centre for Sensors and Electronic Devices (PCSED), Advanced Materials and Nano-Research Centre, Najran University, P.O. Box 1988, Najran 11001, Saudi Arabia; faharraz@nu.edu.sa; 4 Nanomaterials and Nanotechnology Department, Central Metallurgical Research and Development Institute (CMRDI), P.O. Box 87 Helwan, Cairo 11421, Egypt

**Keywords:** cyber-physical security, false data injection attacks, machine learning, state estimation, phasor measurement units, smart grids

## Abstract

Cyber-threats are becoming a big concern due to the potential severe consequences of such threats is false data injection (FDI) attacks where the measures data is manipulated such that the detection is unfeasible using traditional approaches. This work focuses on detecting FDIs for phasor measurement units where compromising one unit is sufficient for launching such attacks. In the proposed approach, moving averages and correlation are used along with machine learning algorithms to detect such attacks. The proposed approach is tested and validated using the IEEE 14-bus and the IEEE 30-bus test systems. The proposed performance was sufficient for detecting the location and attack instances under different scenarios and circumstances.

## 1. Introduction

On 13 July 2019, a cyber-attack on the electrical power system in Manhattan, New York, caused electrical power outages across the city. As a result of the attack, electrical power networks were disrupted, and critical services were placed at risk. A similar attack was previously launched against Ukraine on 23 December 2015, which caused a complete blackout of electrical power systems. These devastating attacks are just a couple of the many that have threatened or impacted electrical power systems in recent years [1,2].

Cyber-attackers deliberately manipulate power network data by injecting bad data into the electrical power systems. They do this by deceiving power control engineers into taking wrong actions or decisions. To preserve the reliability of power grids, it is essential to explore advanced techniques to detect the time and location of data manipulation. This detection of bad data can be managed through the state estimation (SE) methodology.

To obtain information on the real-time state of the power grid, it is imperative to exploit the Supervisory Control and Data Acquisition (SCADA) system using state estimators. Therefore, the energy management system (EMS) is adjusted as a consequence of these states and executes various functions such as power flow and contingency analysis. The SCADA gets the power grid measurement through either remote terminal units (RTUs) or phasor measurement units (PMUs). These measurements include power flow, voltage magnitudes, and angles that enable state estimation [3].

**Figure 1:**
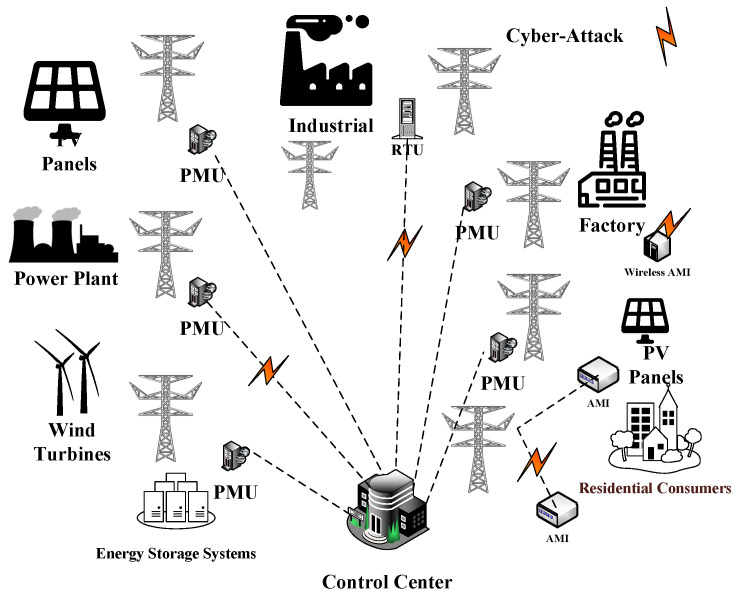
Cyber-threat for smart grids.

Electric power grid metering systems have flourished with phasor measurement units (PMU) and advanced meter infrastructure (AMI). However, these systems have also been attacked in malicious ways (e.g., data manipulation) that have led to the devastation of power grids, as shown in Figure 1. The previously mentioned data manipulation attacks are also referred to as false data injection (FDI) attacks. These attacks have the ability to circumvent bad data detection (BDD) systems, resulting in the electrical power grids initiating but malfunctioning because of the undetected FDI attacks [4].

Typically, DC-estimators are the focus of most FDI literature, as that is where real power flows of RTU measurements are used. In order to appraise the efficacy of state estimation with BDD systems, Teixeira et al. [5] used various FDI attacks. To preserve the power grid measurements versus FDI attacks, an advanced technique was introduced by [6,7]. In [8], the affection of switching network analysis on intense FDI attacks against SE is implemented.

The state estimation of AC power grids is conducted via a nonlinear system. Therefore, it is extremely difficult to avoid the BDD system [9,10]. Consequently, the attackers of power measurements used masks for their FDI attacks [11]. In [12], implementing the wavelet singular entropy (WSE) technique depends on signal processing analysis to detect FDI attacks. Using wireless sensor networks (WSNs), Guan et al. [13] explores the detection of FDI attacks and jamming attacks. An immature FDI structure has been investigated through constructing a forecasting-aided analytics system in [14]. Sparse optimization and low-rank matrix techniques have been utilized to render FDI attacks infeasible using RTUs [15,16]. The majority of these studies were conducted on power grids that utilized RTU meters for the interference of FDI attacks on AC systems.

Phasor measurement units have become highly efficient and have been integrated into various power grid networks in the last decade. This is due to the phasor measurement units’ accuracy and nimble update of the power grid measurements [17]. Thus, several available studies have analyzed the portability of cyber attacks on PMUs. The global positioning system (GPS) approach has been investigated in [18,19]. Designing an innovative mechanism for handling the dilemma of attack vectors on power grids is also assessed in [20]. Alexopoulos et al. [21] have used a vulnerability analysis, in the presence of zero injection buses for launching FDI attacks of PMUs on power networks. Chu et al. [22] examines FDIs physical effects on the N-1 reliable power technology with real-time contingency analysis and a secured power dispatch. Distribution grids are protected from FDI attacks that causes overvoltage using the Convex optimization technique based on second-order cone programming [23]. Ding et al. [24] have develop a bi-level placement model for PMUs placement in the presence of cyber-threats as a defense mechanism. A load redistribution (LR) attack model that utilizes insider threats to power networks is explored by where resources allocation are used by both attackers and system operators Liu et al. [25]. In [26], the phase-locking value (PLV) methodology is implemented to detect FDI attacks, where this approach can only determine the instances of cyber-attack without determining the location. Huang et al. [27] has improved a technique previously used to defend against coordinated cyber-physical attacks (CPAs) based on reducing the number of PMUs.

RTU-based FDI studies are prevalent in literature, whether these units are dedicated to AC or DC estimators [9]. Many researchers have opted to secure RTUs against FDI attacks by strategically deploying PMUs [20]. Others have used PMU data streams along with load forecasts to defend against RTU-based FDI attacks [28].

The main contributions of this work are summarized as follows:

- RTU-based FDI attacks are prevalent in the literature, where compromising several RTUs is necessary for launching successful FDI attacks. As for PMUs, compromising one PMU is sufficient. This work addresses PMU-based FDI attacks.
- It presents an effective approach for detecting FDIs attacks’ moving averages and correlation with several machine learning algorithms.
- The proposed approach is able to identify both the location (targeted PMUs) and the time of the attacks.
- The proposed approach is practical regardless of the window size choice.

The rest of the paper is organized as follows. Section 2 describes state estimation in the presence of PMUs. Section 2.1 discusses the attack strategy for FDI. Section 3 presents the proposed detection mechanism. Section 4 presents the simulation results, and Section 5 concludes the paper.

## 2. Preliminaries

The PMUs and RTUs measurement are used by state estimators to predict the magnitudes and angles of the grid ($\hat{x}$). By making the grid completely observable using PMUs, the nonlinear state estimation can become a linear process [3,29]. One of the main advantages of PMUs is their ability to measure the voltage and current in complex forms. This ability simplifies the state estimation (SE) process and makes it linear. For a system with *N* buses under PMU-based SE, the measurement vector can be expressed as follows:

(1) $$z_{n}=\left[\begin{array}{c} v_{n}^{real}\\ v_{n}^{imag.}\\ i_{n_{k}}^{real}\\ i_{n_{k}}^{imag.} \end{array}\right]=\left[\begin{array}{c} \left|V_{n}\right|\cos\theta_{n}\\ \left|V_{n}\right|\sin\theta_{n}\\ \left|I_{n_{k}}\right|\cos\theta_{nk}\\ |I_{n_{k}}|\cos\theta_{nk} \end{array}\right]$$

where $z_{n}$ is the measurement vector for bus *n*. $|V_{n}|$ and $\theta_{n}$ are the voltage and phase angle for bus *n*. The current flow between buses *n* and *k* is represented by $I_{nk}$. The whole system measurements can be written as follows:

(2) Z(t)=H(x(t))+w(t),

where $Z={\left[z_{1},z_{2}\cdots z_{n}\right]}^{T}$ which represents the measurements for the whole grid. *w* is the noise due to the environment and the sensors. The measurement function H of the grid state vector (x) which is the bus voltages. There are several approaches for estimating the state vector **x**. One of the common approaches is weighted least square which adjusts the measurements’ weights to have the best estimate of x [3,29].

(3) $$\min J\left(x\right)={\left(Z-H\left(x\right)\right)}^{T}R^{-1}\left(Z-H\left(x\right)\right)$$

where *R* is a diagonal covariance matrix whose elements are composed of measurement error variance $\sigma^{2}$. By minimizing J(x) in (3), the best estimates of *x* can be obtained. Minimizing (3) is usually done in an iterative process and at the desired tolerance the grid states can be expressed as

(4) $$\hat{x}={\left(H^{T}R\;H\right)}^{-1}H^{T}R\;Z.$$

For PMU-based state estimation, the SE process can be linear as the measurement and the states vectors are arranged in rectangular forms. The measurement function becomes a composition of the identity matrix and admittance matrix elements corresponding to the current measurements as in (Section 2). Thereby making state estimation a non-iterative process.

(5) $$H=\left[\begin{array}{c} I_{\mathrm{mv}\times 2n}\\ h_{\mathrm{mi},\mathrm{mi}\times 2n} \end{array}\right]$$

where, **mv** and **mi** are the number of voltage and current measurements respectively. The h represents the branch admittance $Y_{ij}$ in a decomposed form such the real and imaginary parts of the branch current $I_{ij}$ are produced separately using the model described above the states $\hat{x}$ can be determined using weighted least square as follows:

(6) $$\hat{x}={\left(H^{T}R^{-1}H\right)}^{-1}H^{T}R^{-1}Z;$$

where *R* is the covariance matrix of the noise.

### 2.1. Attack Model

This subsection describes FDI attacks for smart grids based on PMUs. As the control center conducts state estimation using (6), the adversaries aim to falsify the measurements in (2) without detection. This falsification if not detected will lead to the wrong state estimation, thereby leading to wrong operational decisions by the operators such as overloading or tripping transmission lines. However, falsifying measurements cannot be done arbitrarily; As state estimators, use techniques such as the Chi-square test and Largest normalized Residual (LNR) which can detect abnormal or manipulated measurements [3]. Therefore, for the adversaries to be successful, such attacks need to be masked using the grid topology and avoid detection by BDD or LNR  [8,29,30]. Avoiding detection can be done by building the attack vector $a_{v}$ using the grid information, which can be obtained by a disgruntled employee or by monitoring the data streams (PMU measurements) [26].

(7) $$a_{v}=c\times {\left[0\cdots h_{1}h_{2}\cdots h_{i}\;0\cdots 0\right]}^{T},$$

where

*a^v^* is the attack vector,

*c* is the desired change by the adversary to the true states, 

*h_i_* ∈ **H**.

This attack vector $a_{v}$ is built using several measured signals. For RTU-based FDI, several RTUs need to be compromised [8,29]. However, for PMUs, compromising one PMU can be sufficient as each PMU measures the bus voltage and all current streams of adjacent buses. Therefore, the measurement vector in (2) can be changed as follows:

(8) $$Z_{comp.}=Z+a_{v};$$

where

*a^v^* is the attack vector,

$Z_{\mathrm{comp}.}=[z_{{\mathrm{tru}}_{1}}z_{{\mathrm{tru}}_{2}}\ldots z_{{\mathrm{comp}}_{1}}z_{{\mathrm{comp}}_{2}}\ldots z_{{\mathrm{comp}}_{i}}z_{{\mathrm{tru}}_{{}_{i+1}\ldots }}z_{N}{]}^{T}.$

By using the attack vector in (7) and (10), the LNR is not affected thereby making such attacks undetectable using traditional approaches. If the LNR in (9) is above a certain threshold, the data is flagged and removed. The key aspect of this manipulation is to make the LNR of the compromised measurement as close to the LNR of the original measurements as possible [9].

(9) $$\begin{array}{c} LNR\leq \tau \\ =∥Z-H\hat{x}∥\leq \tau \end{array}$$

(10) $$\begin{array}{c} LNR_{comp.}=∥z_{comp.}-H{\hat{x}}_{comp.}∥=∥z_{tru}+a_{v}-H\left(\hat{x}+c\right)∥\\ =∥z_{tru}+\mathrm{hc}-H\hat{x}-\mathrm{hc}∥=∥z_{tru}-h\hat{x})∥\leq \tau .\\ LNR_{comp.}≅LNR \end{array}$$

Therefore, if the attack vector is constructed as in (10), the FDI will be successful. The adversaries can monitor the measurement data to obtain partial information about the grid and use PMUs for such attacks. Compromising one PMU is enough to construct such a vector and manipulate several states of the grid.

## 3. Methodology

The PMU measurement data is undergone through several pre-processing steps, before feeding measurement data (data streams) to the machine learning algorithms. As using raw data directly or irrelevant feature processing or may add redundancy, which can deteriorate the performance or lead to false classifications. The raw data streams are used to compute the features, using moving average in (11), thereby reducing the fluctuations and noise in the data and to make the streams more stable.

(11) $$\begin{array}{c} X_{M}=\frac{1}{w}\sum_{n=0}^{w}x_{raw}\\ Y_{M}=\frac{1}{w}\sum_{n=0}^{w}y_{raw} \end{array}$$

where,

*w* window size 

*x_raw_* identify raw PMU measurement data

*y_raw_* binary flags identifying attacked samples

*X_M_* identifies moving average of the measurements *Z* in (2) and (8).

*Y_M_* identifies moving average ground truth (which measurements are attacked).

It should be noted that the $x_{raw}$ in the above equation is the data obtained by the PMUs through their channels is referred to as *Z* in (2) and (8).

**Figure 2:**
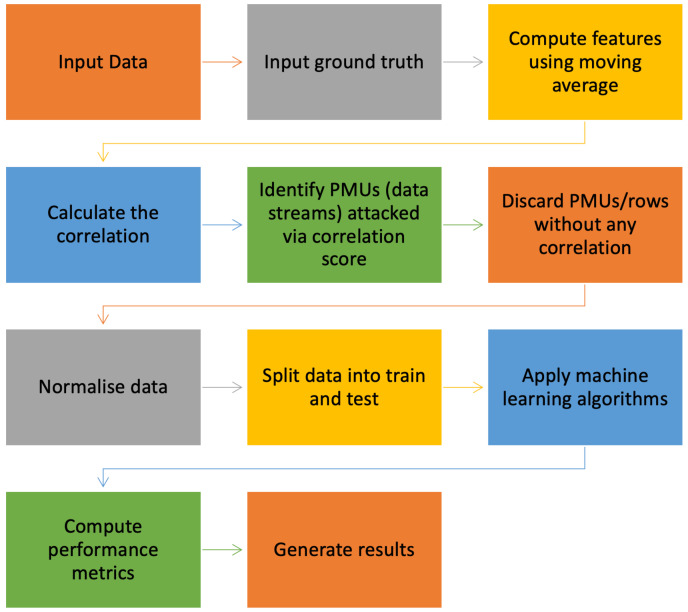
Detection of False Data Injection using machine learning.

The next step of the pre-processing is to compute the Pearson correlation between all the different data streams recorded to find the most correlated measurement data (data streams) as in (12). The correlated streams are the streams of the measurement data that are of interest since these are the ones that were affected by the FDIs. A supervised learning approach is used where the data streams are split into different parts to model, then test and validate. In supervised learning, the desired results (classification) are referred to as the ground truth. The ground truth is used to help model the system in the initial stage and used in the other stages to validate and test the performance of the model [31]. These correlated data streams (measurement data) are further compared with the ground truth values to validate if the correlation process has identified the accurate data streams under attack due to the FDIs. After the accurate identification of the attacked PMU channel via correlation, the data streams are further fed into the machine learning algorithms for classification purposes. Flow chart of all the processing steps opted to detect the rows (attack location) and the attacked samples of attacked PMU are presented in Figure 2.

(12) $$C_{r}=\frac{\Sigma_{M}\left(\left(X_{M}-\mathrm{mean}\left(X_{M}\right)\right)\left(Y_{M}-\mathrm{mean}\left(Y_{M}\right)\right)\right)}{\sqrt{\sum_{M}{\left(X_{M}-\mathrm{mean}\left(X_{M}\right)\right)}^{2}\sum_{M}{\left(Y_{M}-\mathrm{mean}\left(Y_{M}\right)\right)}^{2}}}$$

where,

*X_M_* identifies moving average

*Y_M_* identifies ground truth

*C_r_* identifies correlation between *X_M_* and *Y_M_*

### 3.1. Ground Truth Adjustment

The original ground truth (the true classification) $y_{raw}$ obtained to validate the classifier performance is also updated as per the window size length that moving average filter uses in (11). This is important to keep the equal number of measurement samples in the dataset obtained after the moving average stage and in the transformed ground truth. To achieve this, majority voting criteria is used in the ground truth transformation which means that the majority class will be sustained in the transformed ground truth data $X_{M}$ and $Y_{M}$. For example, if the window length of moving average is 5 and we have $y_{raw}$ = [1,1,1,0,0]. Then, this scenario will generate ground truth value of $Y_{M}$ = [1] since the “1” is the majority class in the $y_{raw}.$

### 3.2. Machine Learning Algorithms for the Detection of False Data Traces

The data streams of the PMUs obtained after the moving average are then applied to the different machine learning algorithms to detect the false data injections and their corresponding time instances. Three machine learning algorithms are used. We assume the adversaries are targeting the minimum number of measurements that guarantees a successful attack. Targeting a high number of measurements is taxing on the resources of the adversaries and raises the risk of detection [9,26,27]. Therefore, the measurements data (data streams) are highly skewed, since only a minority of the data is manipulated by the adversaries. Therefore, when evaluating the performance of the algorithms F-score is preferred over the other performance measures [32]. The expression to compute F-score is presented in (13).

(13) $$F_{1}\;\mathrm{score}=\frac{2TP}{2TP+FP+FN}$$

where

*TP* normal samples identified correctly (true positive)

*FP* attacked samples identified incorrectly (false positive)

*TN* attacked samples identified correctly (true negative)

*FN* normal samples identified incorrectly (false negative)

### 3.3. Support Vector Machine

One of the machine learning algorithms used to detect the FDIs is the support vector machines (SVM). The SVM is widely used for classification problems [33,34] in a variety of different domains due to their high predicting power, high margin and the use of the support vectors to better fit the data and their capabilities of handling the data with outliers [33]. Therefore, SVM is quite efficient machine learning algorithm, easy to understand, implement and interpret. Each object for classification is represented as a point in an n-dimensional space and the coordinates of this point are usually called features. SVMs perform the classification task by drawing a hyperplane that is a line in 2-D or a plane in 3-D in such a way that all points in one category are on one side of the hyperplane and all points of the other category are on the other side. There could be multiple hyperplanes and SVM tries to find the one that best separates the various categories in the sense that it maximizes the distance to points in either category. This distance is called the margin and the points that fall exactly on the margin are called the supporting vectors. In this study, we have implement SVM classifier since we have discrete output classes to classify i.e., attacked vs non-attacked. The SVM regressor is also used to classify the continuous variables. The mathematical expression to compute margin for the linear SVM [31] is shown in (14).

(14) $$\mathrm{argmin}=\mathrm{argmin}d\left(x\right)=\mathrm{argmin}\frac{\left|x\times w+b\right|}{\sqrt{\sum_{i=1}^{d}w_{i}^{2}}}$$

where *x* is the training data vector, *d* is the margin of separation between hyperplanes, *w* is the weight vector and *b* is the constant. The SVM is implemented in python using the sklearn libraries and with the linear kernel and the complexity is set to 1.

The SVM classification algorithm was developed by setting the various parameters as given in Table 1. The kernel cache size is set to 200 which acts as a buffer. Degrees is set to 1 and linear kernel function is used in this study. The linear kernel served the purpose in this study, therefore more complex kernel methods such as polynomial, radial basis function or sigmoid were not further explored. As there are binary-classes that’s why ’ovr’ has been used which mean one vs rest decision function. The mathematical expression (13) was used to compute the margin for the linear SVM [32].

### 3.4. Extreme Gradient Boosting Classifier

Ensemble methods are quite useful in combining the results obtained from several individual estimators together to improve the system performance. The combined performance of estimators is preferred instead of using individual estimator’s based prediction that can result into lower accuracy. The eXtreme Gradient Boosting (XGB) classifier is type of ensemble classifiers used in this study to detect the FDI. This XGB is relatively a new algorithm and has been frequently used in different domains [34,35] due to the improvisation it has achieved over the standard GB classifier through the efficiently execution and implementation of the approximation methods. Like every classifier, XGB also got a mechanism to find the optimal parameters by optimizing the regularized objective function shown in (15) [36]    

(15) $$L^{\left(t\right)}=\sum_{i=1}^{n}l\left(y_{i},{\hat{y}}_{i}^{\left(t-1\right)}+f_{t}\left(x_{i}\right)\right)+\Omega \left(f_{t}\right)$$

where $L^{\left(t\right)}$ is objective function, *n* is the number of examples in the training dataset, *t* is the *t*th iteration of the tree, *i* is the *i*th instance of the training example, $x_{i}$ is the features value at the instance i, $y_{i}$ is the actual value, ${\hat{y}}_{i}$ is the predicted value of the ensemble tree, $f_{t}$ represents the *t*th tree iteration, Ω(ft) is the measure of model complexity,. The XGB is implemented in python and the simulations are set as follows i.e., booster = gb tree, minimum child weight 1, learning rate 0.3, gamma 0, maximum depth 6. Table 2 summarizes the classification parameters.

### 3.5. Quadratic Discriminant Analysis

The third classifier used to detect FDI is quadratic discriminant analysis (QDA). As is evident from the name, the QDA generates the quadratic decision boundaries to train the classifier. Such classifiers are preferred due to their less computational requirements and also not requiring the hyper parameters tuning.The QDA is also implemented in Python. The expression to compute the estimated class x(C(x)) using LDA [37] is presented in (16).

(16) $$C\left(x\right)=\underset{k}{\mathrm{argmax}}\delta_{k}\left(x\right)$$

where C(x) is the estimated class, *x* is the dataset instance, *k* is the number of classes, $\delta_{k}\left(x\right)$ is the quadratic discriminant function.

## 4. Simulation and Results

**Figure 3:**
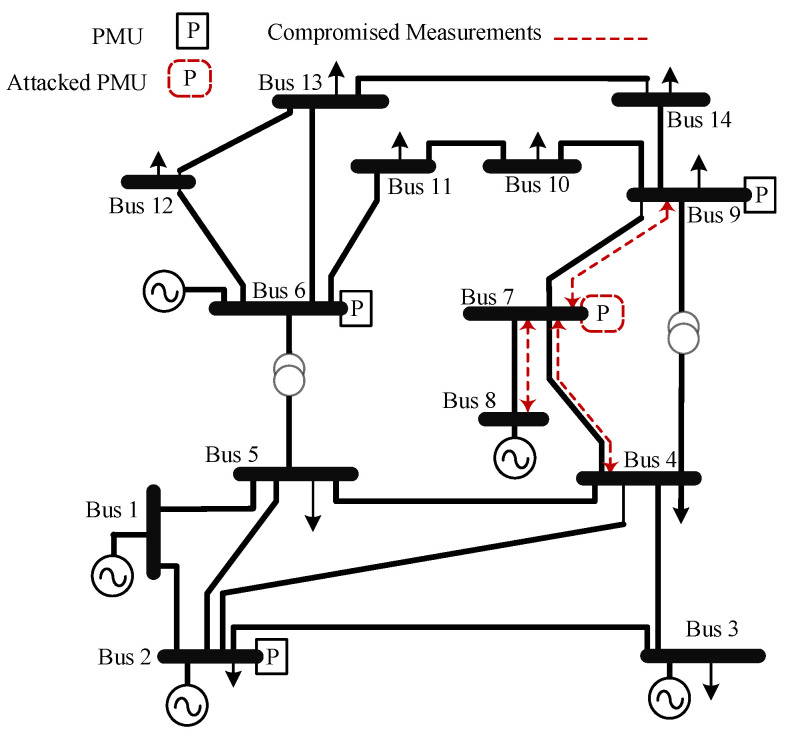
IEEE 14-bus test system [26].

**Figure 4:**
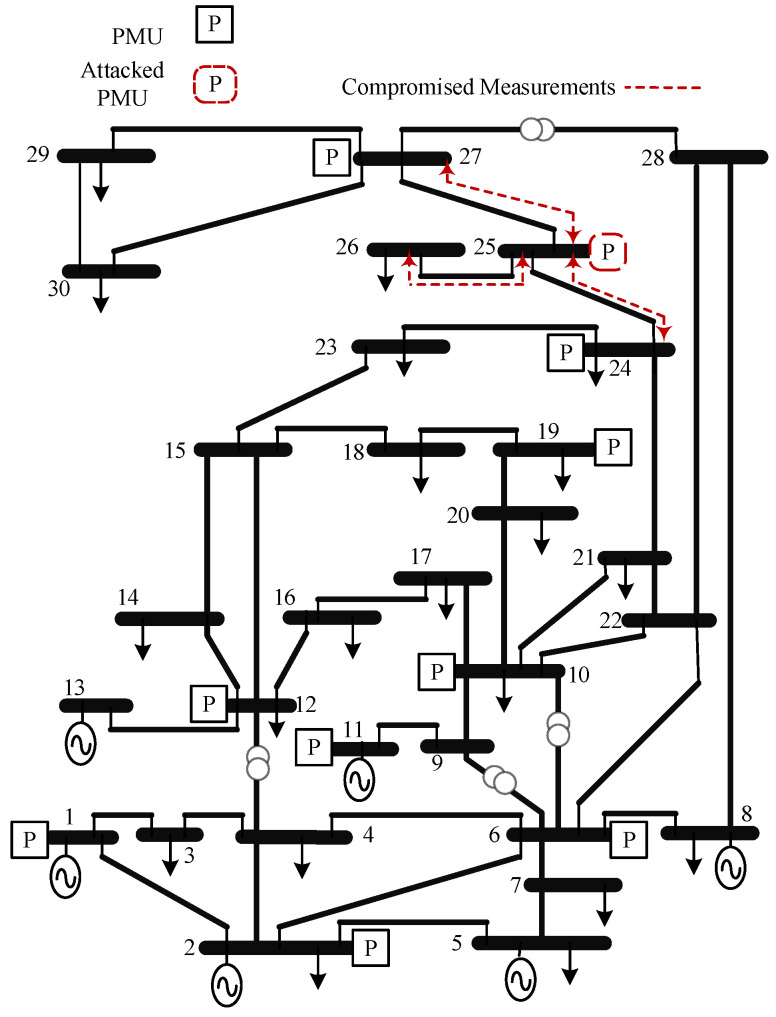
IEEE 30-bus test system [26].

This section presents the ML approach for detecting PMU-based FDIs. The approach is tested on the IEEE 14-bus and the IEEE 30-bus test systems. The PMUs are deployed for both systems to achieve complete observability [38,39,40], as shown in Figure 3 and Figure 4. The FDIs are tested on both systems using the approach mentioned in Section 3 which is summarized in Algorithm 1. In the proposed approach, PMUs are assumed to measure the signals at 30 sample per second, where each PMU measures the current flow of all bordering buses.
**Algorithm 1:** FDI detection algorithm 
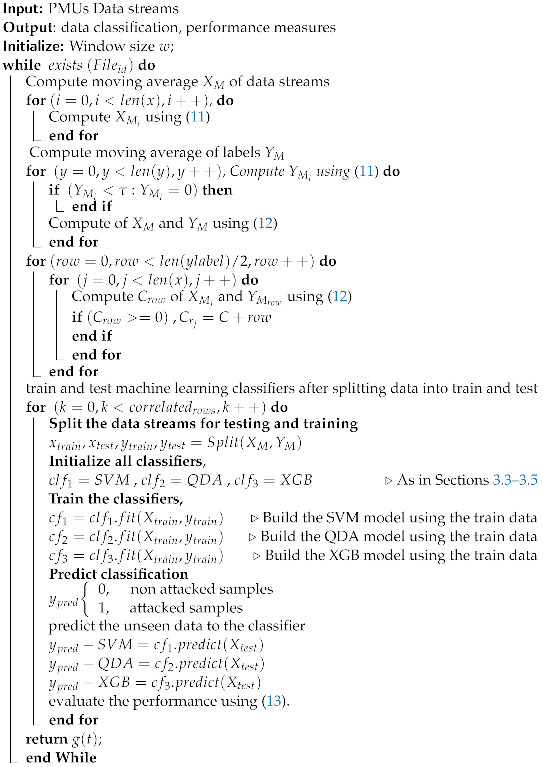


### 4.1. Case Studies

To test the validity of our approach, several scenarios are tested for both systems. The load profile is varied, and 50 Monte Carlo simulations are used for each scenario. Four scenarios are used as follows:

- **Scenario I:** In this scenario, the time and duration of the attack is randomized for all Monte Carlo simulations. However, the attack intensity and location are kept constant.
- **Scenario II:** In this scenario, the time and duration of the attack is randomized for all Monte Carlo simulations. The location of the attack is also randomized *per each simulation.* However, the attack intensity is kept constant.
- **Scenario III:** In this scenario, the time and duration of the attack is randomized for all Monte Carlo simulations. The attack intensity $a_{v}$ and location are varied *per each simulation.*
- **Scenario IV:** In this scenario, Multiple random PMUs are attacked simultaneously; the attack vector *a* changes randomly for each Monte Carlo simulation. The duration of the attack is also randomized.

The above mentioned scenarios are summarized in Table 3. We have conducted several experiments by varying the size of the moving average window in (11) from 2 to 12 as these best fits in the scenario generated. Our findings suggested that a change in the window size does not affect the performance of the machine learning-based method in detecting the false data streams. This is true in both cases, when findings the location of the attacked bus via correlation as well as detecting the data samples of the attacked samples using machine learning algorithms. These findings are quite encouraging and suggest that the proposed system is capable of detecting the attacked PMUs and their time matter, regardless of the length of feature window used during the moving average filter processing. The window length is kept to “5” in all the upcoming scenario to maintain the uniformity among the results.

### 4.2. Attacked Bus Detection

The performance analysis of the proposed hybrid (correlation and machine learning base approach) false data injection method is presented in Table 1. The column “cases” presents the different dataset scenarios, the attacked PMU shows the detected location of the PMU, the accuracy of location shows the difference between the actual false data injection identified through the I-Flag data flag and the predicted location detected through the proposed false data injected detection method. The last column presents the performance of machine learning classifiers in detecting the accurate data samples or samples when the attacked is occurred. It is quite evident from Table 4.

### 4.3. Time Stamp Prediction Using Machine Learning

**Figure 5:**
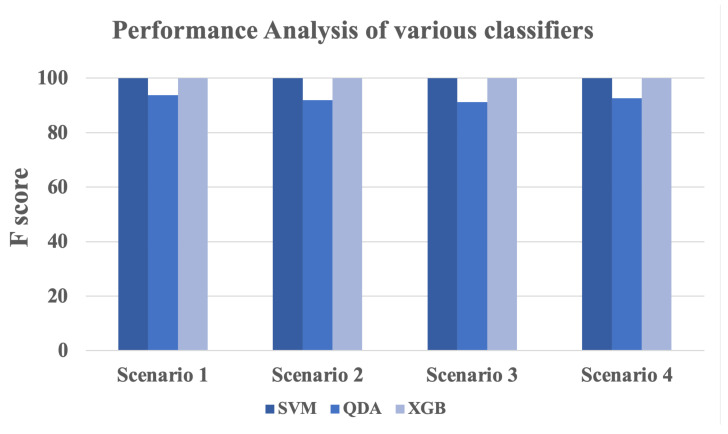
Performance analysis of various classifiers and data collection scenarios.

Time stamp prediction of attacked vs non-attacked samples is accomplished by developing three classifiers on the FDI dataset. The overall performance is obtained by the three classifiers (SVM, QDA, XGB) using all the three dataset scenarios is presented in Figure 5.

It is quite obvious from the Figure 5 that most of the classifiers (2 out of 3) implemented in this study achieved an overall performance (F ${}_{1}$ score) of 100% which is quite encouraging and show the strength of the proposed system in classifying attacked vs non-attacked data streams. More results and discussion are presented in the upcoming Section 4.4, Section 4.5, Section 4.6 and Section 4.7.

### 4.4. Scenario I

The performance analysis of three classifiers implemented in this study is presented in the form of confusion matrix in Table 5 along with the F-score. Its is quite evident from the results that SVM and XGB are able to detect the attacked and non-attacked samples with very high performance of 100% followed by QDA with a performance of above 93%. These results showed the strength of the proposed novel machine learning method in detecting the data samples of false injected data in the network.

### 4.5. Scenario II

The results obtained by the machine learning classifies in detecting the false data traces from Mx scenario are presented in Table 6. The findings of the scenario II are very positive and depicted that SVM and XGB are capable of detecting the false data data samples with F-score of 100%. The QDA also performed well and achieved performance of around 92% in detecting the false data streams.

### 4.6. Scenario III

The performances and confusion matrices obtained in the scenario III using the IEEE-30 bus dataset are presented in Table 7. Though this scenario is denser and use more buses in the network. Still the proposed machine learning based methods are capable enough to detect the false data injection streams with F-score of 100%, succeeded by QDA with F-score of 92.14%.

### 4.7. Scenario IV

The performances and confusion matrices obtained in the scenario IV using the IEEE-30 bus dataset are presented in Table 7. Though this scenario is denser and use more buses in the network. Still the proposed machine learning based methods are capable enough to detect the false data injection streams with F-score of 100%, succeeded by QDA with F-score of 92.88%.

It is quite evident from the findings presented in Table 4, Table 5, Table 6, Table 7 and Table 8 that SVM and XGB outperformed the QDA classifier. This could be due to the fact that both of the best performing machine learning classifiers (SVM and XGB) not only find the optimal solution that provides the best fit for the data but also restricts the machine learning classification model from overfitting. This enables these classifiers to achieve better performance not only on the training set but also on the test sets. On the other end, QDA does not incorporates optimization inside the machine learning model and assumes that data comes from normal distribution which is not often the case when the data samples are taken from relatively small cohort or subset.

A comparative study is presented in Table 9, where our approach is compared with the PLV approach of [26]. By checking the F score for detecting attacks, the QDA of our approach performance is lower than all the other approaches, including the phase lock value (PLV). However, the SVM and XGB are the superior approaches, although the margin of improvement is not high. The main advantage of our approach is the ability to identify the attacked PMUs (location of the attack). As for the PLV, although the performance is very high. Their approach is dependent on the choice of the window size; choosing a large window size deteriorates the performance greatly. Moreover, the PLV compares the measurement at one time instant with each other and uses the results to identify the attack time. However, this approach is unable to identify the attacked PMUs. Our approach, on the other hand, uses the Pearson correlation to identify the attacked PMUs and uses the machine learning algorithms to identify the attacked sample.

## 5. Conclusions

This paper introduces a novel approach for detecting FDIs using moving average and correlation along with ML algorithms. The proposed detection mechanism was tested under several scenarios where load profile is varied and FDIs are varied in intensity, location and duration. Using our approach, we were able to detect the location of the attack with a 100% accuracy for all cases. For the system operators, location detection has a higher priority over the detection of the attack timing.

In the proposed approach, the window size does not affect the performance of the machine learning based method in detecting the false data streams. This is true in both cases, when findings the location of the attacked bus via correlation as well as detecting the attacked samples using machine learning algorithms. These findings are quite encouraging and suggest that the proposed system is capable of detecting the attacked PMUs and their time matter, regardless of the length of feature window used during the moving average filter processing.

The main contributor behind the high performances achieved by the XGB and the SVM is the utilization of pre-processing and processing steps introduced by the authors prior to the implementation of classifiers. These are unique contributions of this work where significant data streams are identified through the Pearson correlation, and then the data streams are further processed through the feature engineering stage in the form of windowing and moving average. The feature processing step has helped the classifiers to better distinguish between the attacked versus non-attacked classes. Moreover, the ensemble nature of the XGB classifier with improved and efficient execution and implementation of the approximation methods and the SVM-based maximum margin separation between the hyper-planes also contributed towards the high performances obtained.

One of the future directions is to investigate FDIs in hybrid estimators where both RTU and PMU measurements are used, and the asynchronization of RTUs and PMUs complicates state estimation and opens new vulnerabilities for FDIs.

## Figures and Tables

**Table 1:** SVM classification parameters.

Sr.No	Parameter’s List of SVM
1	cache_size = 200,
2	decision_function_shape = ’ovr’
3	Complexity = 1
4	kernel = linear

**Table 2:** XGB classification parameters.

XGB Parameters	Selected Value
booster	gbtree
learning_rate	0.3
max_depth	6
min_child_weight	1
sampling_method	uniform
lambda	1

**Table 3:** Summary of Scenarios of the FDIs experiments.

Scenarios	Number of Monte-Carlo Simulations	Attacked PMU Location	Attack Vector $a_{v}$	Duration of the Attack
IEEE-14 bus test system				
Scenario I	50	Constant	Constant	Variable
Scenario II	50	A single random PMU	Constant	Variable
Scenario III	50	A single random PMU	Variable	Variable
IEEE-30 bus test system				
Scenario I	50	Constant	Constant	Variable
Scenario II	50	A single random PMU	Variable	Constant
Scenario III	10	A single random PMU	Variable	Variable
Scenario IV	10	Multiple random PMUs	Variable	Variable

**Table 4:** Performance of detecting location of Attacked PMUs.

Case	Attacked PMUs	$a_{v}$	Identification Accuracy
IEEE-14 bus test system			
Scenario I: case 1	7	constant	100%
Scenario I: case 5	2	constant	100%
Scenario II: case 9	6	variable	100%
Scenario II: case 19	9	variable	100%
Scenario III: case 2	7	variable	100%
Scenario III: case 7	6	variable	100%
IEEE-30 bus test system			
Scenario II: case 3	12	variable	100%
Scenario II: case 7	8	variable	100%
Scenario III: case 8	2	variable	100%
Scenario III: case 5	24	variable	100%
Scenario IV: case 2	11, 27	variable	100%
Scenario IV: case 7	1, 12	variable	100%

**Table 5:** Sample of the performance for scenario I.

F-Score	SVM		
100%	Predicted Class		
	Attacked	Not Attacked	← Classified as
Actual Class	4099	0	Attacked
	0	60,700	Not Attacked
**F-Score**	**QDA**		
93.78%	Predicted Class		
	Attacked	Not Attacked	← Classified as
Actual Class	4099	0	Attacked
	786	59,914	Not Attacked
**F-Score**	**XGB**		
100%	Predicted Class		
	Attacked	Not Attacked	← Classified as
Actual Class	4099	0	Attacked
	0	70,700	Not Attacked

**Table 6:** Sample of the performance for scenario II.

F-Score	SVM		
100%	Predicted Class		
	Attacked	Not Attacked	← Classified as
Actual Class	6513	0	Attacked
	0	58,286	Not Attacked
**F-Score**	**QDA**		
91.99%	Predicted Class		
	Attacked	Not Attacked	← Classified as
Actual Class	6513	0	Attacked
	1390	56,896	Not Attacked
**F-Score**	**XGB**		
100%	Predicted Class		
	Attacked	Not Attacked	← Classified as
Actual Class	6513	0	Attacked
	0	58,286	Not Attacked

**Table 7:** Sample of the performance for scenario III.

F-Score	SVM		
100%	Predicted Class		
	Attacked	Not Attacked	← Classified as
Actual Class	5678	0	Attacked
	0	59,121	Not Attacked
**F-Score**	**QDA**		
92.14%	Predicted Class		
	Attacked	Not Attacked	← Classified as
Actual Class	5678	0	Attacked
	968	58,153	Not Attacked
**F-Score**	**XGB**		
100%	Predicted Class		
	Attacked	Not Attacked	← Classified as
Actual Class	5678	0	Attacked
	0	59,121	Not Attacked

**Table 8:** Sample of the performance for scenario IV.

F-Score	SVM		
100%	Predicted Class		
	Attacked	Not Attacked	← Classified as
Actual Class	17,887	0	Attacked
	0	46896	Not Attacked
**F-Score**	**QDA**		
92.88%	Predicted Class		
	Attacked	Not Attacked	← Classified as
Actual Class	17,887	0	Attacked
	1371	45,525	Not Attacked
**F-Score**	**XGB**		
100%	Predicted Class		
	Attacked	Not Attacked	← Classified as
Actual Class	17,887	0	Attacked
	0	46,896	Not Attacked

**Table 9:** Comparison of performance with the literature.

Case	Our Approach SVM	Our Approach QDA	Our Approach XGB	Ref. [26]
IEEE-14 bus test system				
Scenario I: Average F-score	100	93.68	100	99.99
Scenario II: Average F-score	100	92.42	100	99.99
Scenario III: Average F-score	100	92.41	100	99.80
IEEE-30 bus test system				
Scenario I: Average F-score	100	92.11	100	—
Scenario II: Average F-score	100	92.21	100	—
Scenario III: Average F-score	100	92.34	100	98.97
Scenario IV: Average F-score	100	91.89	100	—

## Data Availability

Not applicable.
